# Variation by default: cesarean section discharge opioid prescription patterns and outcomes in Military Health System hospitals: a retrospective longitudinal cohort study

**DOI:** 10.1186/s12871-022-01765-8

**Published:** 2022-07-12

**Authors:** Krista B. Highland, Ian Robertson, Monica Lutgendorf, Germaine F. Herrera, Alexander G. Velosky, Ryan C. Costantino, Michael S. Patzkowski

**Affiliations:** 1 Defense and Veterans Center for Integrative Pain Management, Department of Anesthesiology, Uniformed Services University, 4301 Jones Bridge Road, Bethesda, MD 20814 USA; 2 Henry M. Jackson Foundation for the Advancement of Military Medicine, Inc., 6720A Rockledge Dr., #100, Bethesda, MD 20817 USA; 3 School of Medicine, Uniformed Services University, 4301 Jones Bridge Road, Bethesda, MD 20814 USA; 4 Department of Gynecological and Obstetrics Surgery, Uniformed Services University, 4301 Jones Bridge Road, Bethesda, MD 20814 USA; 5 Enterprise Intelligence and Data Solutions (EIDS) Program Office, Program Executive Office, Defense Healthcare Management Systems (PEO DHMS), San Antonio, TX USA; 6 Department of Military and Emergency Medicine, School of Medicine, Uniformed Services University, 4301 Jones Bridge Road, Bethesda, MD 20814 USA; 7 Department of Anesthesiology, Brooke Army Medical Center, 3551 Roger Brooke Drive, Fort Sam Houston, TX 78234-6200 USA

**Keywords:** Cesarean section, Delivery, Labor, Opioid, Pain, Pregnancy, Healthcare Variation, Prescribing Practices, Health Services Research, Non-Opioid Pain Medication

## Abstract

**Background:**

To examine factors associated with post-Cesarean section analgesic prescription variation at hospital discharge in patients who are opioid naïve; and examine relationships between pre-Cesarean section patient and care-level factors and discharge morphine equivalent dose (MED) on outcomes (e.g., probability of opioid refill within 30 days) across a large healthcare system.

**Methods:**

The Walter Reed Institutional Review Board provided an exempt determination, waiver of consent, and waiver of HIPAA authorization for research use in the present retrospective longitudinal cohort study. Patient records were included in analyses if: sex assigned in the medical record was “female,” age was 18 years of age or older, the Cesarean section occurred between January 2016 to December 2019 in the Military Health System, the listed TRICARE sponsor was an active duty service member, hospitalization began no more than three days prior to the Cesarean section, and the patient was discharged to home < 4 days after the Cesarean section.

**Results:**

Across 57 facilities, 32,757 adult patients had a single documented Cesarean section procedure in the study period; 24,538 met inclusion criteria and were used in analyses. Post-Cesarean section discharge MED varied by facility, with a median MED of 225 mg and median 5-day supply. Age, active duty status, hospitalization duration, mental health diagnosis, pain diagnosis, substance use disorder, alcohol use disorder, gestational diabetes, discharge opioid type (combined vs. opioid-only medication), concurrent tubal ligation procedure, single (vs. multiple) births, and discharge morphine equivalent dose were associated with the probability of an opioid prescription refill in bivariate analyses, and therefore were included as covariates in a generalized additive mixed model (GAMM). Generalized additive mixed model results indicated that non-active duty beneficiaries, those with mental health and pain conditions, those who received an opioid/non-opioid combination medication, those with multiple births, and older patients were more likely to obtain an opioid refill, relative to their counterparts.

**Conclusion:**

Significant variation in discharge pain medication prescriptions, as well as the lack of association between discharge opioid MED and probability of refill, indicates that efforts are needed to optimize opioid prescribing and reduce unnecessary healthcare variation.

**Supplementary Information:**

The online version contains supplementary material available at 10.1186/s12871-022-01765-8.

## Background

Cesarean section (CS) rates have risen dramatically in the last two decades, accounting for 20% of live births globally and over 30% of live births in the United States [1, 2]. The increasing prevalence of CS is especially important to note given the association with significant postoperative pain [3] and potential linkage to the ongoing opioid crisis in the United States [4]. Adequate pain management after CS is crucial both during the acute stages of postoperative recovery and in the prevention of persistent postoperative pain [5].

The American College of Obstetricians and Gynecologists (ACOG) and PROSPECT guidelines recommend a multimodal pain management approach to include pharmacologic agents such as non-steroidal anti-inflammatory drugs (NSAIDS), acetaminophen, and opioids. In particular, parenteral or oral opioids should only be used as a “rescue” method when analgesia from neuraxial anesthesia and nonopioid adjuncts is inadequate [6, 7]. Despite these recommendations, one study demonstrated that over 75% of patients filled peripartum opioid prescriptions containing 40 opioid tablets, on average, of which 20 opioid tablets were used; however, 95% of patients had not disposed of excess medication at time of interview [8]. Such a high prevalence of excess opioid medication puts patients at risk of acute adverse opioid-related events such as respiratory depression and sedation, and also the risk of persistent opioid use, dependence, and diversion [9]. Given these risks, non-opioid adjuncts such as NSAIDs and acetaminophen are recommended as first-line analgesics to reduce the need for opioids and related effects of opioid-centric pain management (e.g., drowsiness and sedation) after CS deliveries [10].

The goal of our study was to examine factors associated with post-CS analgesic prescription variation at hospital discharge in patients who are opioid naive (no opioid prescription dispense event in the previous six months), and to examine the relationship between pre-CS factors (including patient- and care-level factors) and discharge opioid prescribing on post-CS outcomes (e.g., probability of 30-day post-CS opioid refill) across a large healthcare system. We hypothesized that the probability of an opioid refill within 30 days after CS would be associated with demographic and health characteristics, medical care (e.g., receipt of various classes of medications before CS), discharge opioid prescription dose and type (e.g., opioid/non-opioid combination medication with a non-opioid vs. opioid-only), discharge non-opioid prescription receipt, and healthcare facility.

## Methods

### Data sources and record selection

The Walter Reed National Military Medical Center Institutional Review Board (WRNMMC-EDO-2020–0560) provided an exempt determination, waiver of consent, and waiver of HIPAA authorization for research use in the present retrospective longitudinal cohort study. All methods were carried out in accordance with relevant guidelines and regulations under Ethics approval and the Helsinki Declaration. Patient records were included in analyses if: sex assigned in the medical record was “female,” age was 18 years or older, the CS occurred between January 2016 to December 2019 in the Military Health System (MHS), the listed TRICARE sponsor was an active duty service member, hospitalization began no more than three days prior to the date of CS, and the patient was discharged to home within three days after the CS date (maximum inpatient stay was seven days). Lastly, patient records were excluded if the patient was hospitalized in the month prior to or after the CS, received a morphine equivalent dose (MED) greater than the 99th percentile of MEDs (e.g., outliers), received an opioid prescription within the 6-month period prior to the CS, or received more than one opioid prescription at discharge. All data was extracted from the MHS Data Repository.

### Variables of interest

#### Patient- and care-level characteristics

Demographic characteristics included age, race and ethnicity, and beneficiary type (active duty versus family member). The facility of the CS was extracted. Additional care-level information included the presence of a concurrent tubal ligation procedure.

#### Medications

Pharmacy transaction records were extracted for the following types of medications, per American Hospital Formulary Service classification: opioid agonists and non-opioid pain medications (NSAID and acetaminophen). Opioid prescription information included pill quantity, days supply, the total oral morphine equivalent dose (MED) for the prescription, the morphine equivalence (MEQ) for each pill, and whether it was an opioid-only versus opioid/non-opioid combination medication.

#### Diagnoses

Documented mental health, substance use disorders, alcohol use disorders, and musculoskeletal pain conditions within the 6-month period prior to CS were consolidated into four variables. Mental health ICD10 codes included F43.2 (adjustment disorder), F40-F42 (anxiety), F30-F39 (mood), and F43.1 (post-traumatic stress); substance use disorders included F11-F16 and F18, F19; and alcohol use disorders included F10. Musculoskeletal pain conditions included the following ICD10 codes: M50-M54 (spinal conditions); M25.5 (joint pain); M15-M19 (osteoarthritis); S22, S42, S52, S62, S72, S82, and S92 (bone fracture); S13, S23, S43, S53, S63, S73, S83, and S93 (dislocation or sprain); S16, S46, S56, S66, S76, S86, and S96 (muscle or tendon injury). Additional diagnoses corresponded to those related to sexually transmitted infection (O98.311—O98.33), preeclampsia (O11.2, O11.3, O11.9, O14.00, O14.02, O14.03, O14.10, O14.12, O14.13, O14.90, O14.92, O14.93), premature labor (O60.10X0, O60.12X0, O60.13X0), gestational diabetes (O24.410, O24.414, O24.415, O24.419), hypertension (O10.011—O10.019, O10.911—O10.919, I10), chorioamnionitis (O41.1010—O41.1299), single birth delivery (Z37.0), and postpartum depression (O90.6) were also extracted.

#### Outcome

The primary outcome was the probability of receiving a second (refill) opioid prescription (yes/no) within 30-days after the CS.

### Analytic Plan

Univariate statistics (e.g., means and standard deviations, medians and 25th/75th percentiles, frequencies and percentages) were used to describe care- and patient-level variation in covariates. The *comparegroups* R package [11] was used to create the descriptive table. Second, bivariate (e.g., non-parametric correlations, Kruskal–Wallis sum rank tests) analyses examined the relationship between pre-CS patient- and care-level factors (e.g., age, days hospitalized, mental health conditions, pain conditions, substance and alcohol use disorders, gestational diabetes, etc.) with discharge MED and opioid refill. Factors significantly associated with discharge MED and refill in bivariate analyses were included as covariates in a multivariable generalized additive mixed model (GAMM). The primary outcome of the GAMM was the probability of opioid refill within 30 days after CS and included records of patients who received an opioid prescription at discharge. The GAMM included a random effect for the facility. The GAMM was estimated using the *gamm4* R package [12]; marginal means were computed using the *ggeffects* R package [13]; the random effect of treatment facility was extracted using the *arm* R package [14]; and plots were constructed using the *ggplot2* [15] and *ggpubr* [16] R packages. Sensitivity analyses were conducted such that the GAMM was repeated for active duty service members and family members separately. Statistical significance was indicated by *p* < 0.05.

## Results

### Univariate statistics

**Fig. 1:**
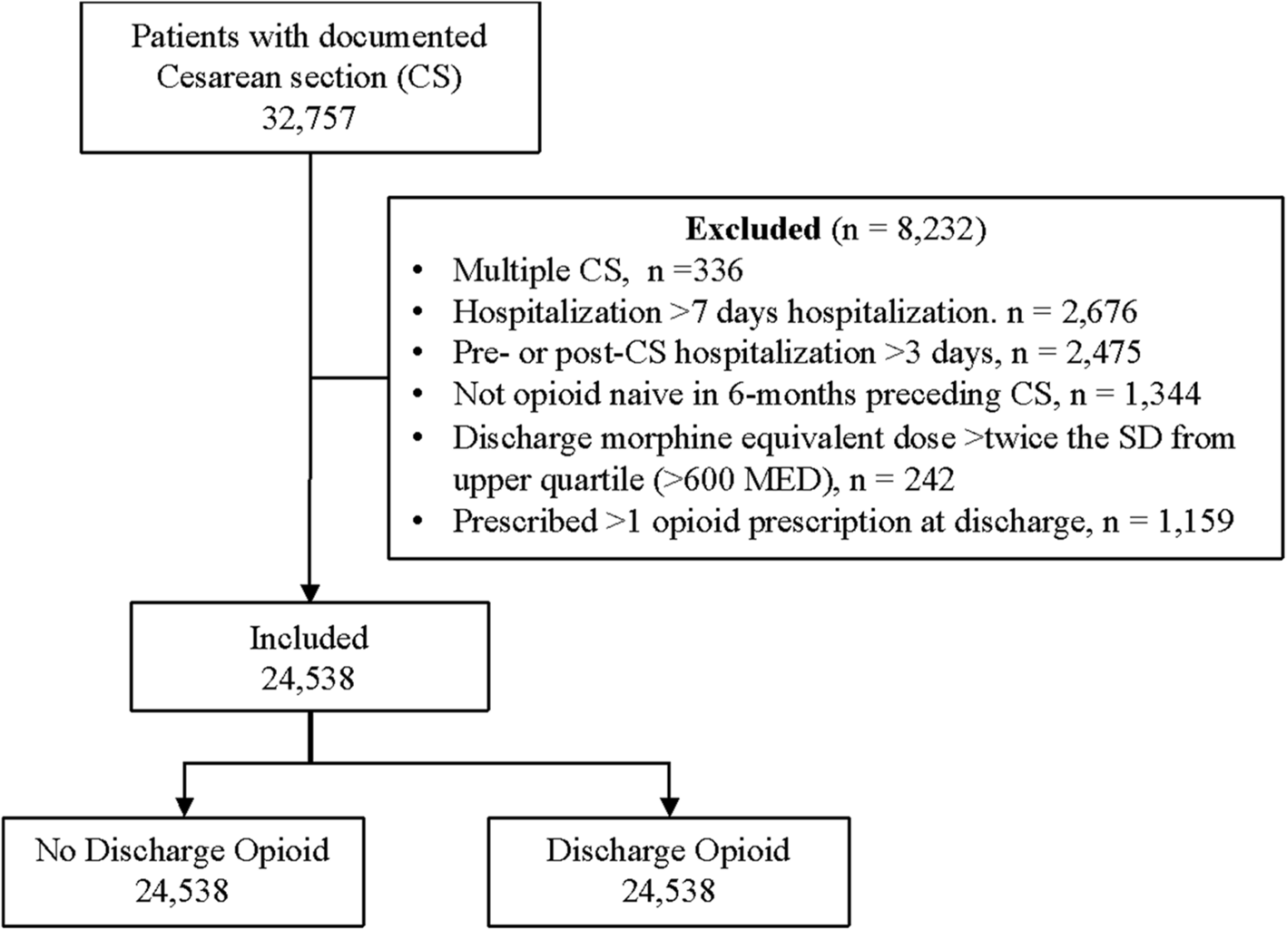
Flow diagram

In the study period, 32,770 adult patients had a single documented CS procedure, of whom, 24,557 met inclusion criteria and were used in the analyses (Fig. 1). Descriptive statistics are reported in Table 1. Most patients (92.6%) received an opioid/non-opioid combination medication and a separate non-opioid pain medication. Approximately 5.2% of the sample did not receive an opioid prescription at discharge (Supplemental Figure 1). Of these patients, 15% received an opioid prescription in the first month following the CS (Table 1).

**Table 1:** Descriptive statistics of the full sample, those who did not receive an opioid at discharge, and those who did not and did refill an opioid prescription within 30 days after discharge

	Full Sample	No Discharge Opioid	No Refill*	Refill*	*p*-value*
	(*N* = 24,557)	*n* = 1,274 (5.2%)	*n* = 21,740 (88.5%)	*n* = 1,543 (6.3%)	
**Demographic and Pre-Cesarean-Section Factors**					
Age, median years [IQR]	28.0 [24.0;32.0]	29.0 [24.0;33.0]	28.0 [24.0;32.0]	29.0 [25.0;33.0]	< 0.01
Race and Ethnicity, n (%)					0.62
Latinx white	851 (3.5%)	31 (2.4%)	774 (3.6%)	46 (3.0%)	
Non-Latinx Asian	1,437 (5.9%)	66 (5.2%)	1,280 (5.9%)	91 (5.9%)	
Non-Latinx Black	4,306 (17.5%)	206 (16.2%)	3,847 (17.7%)	253 (16.4%)	
Non-Latinx white	12,221 (49.8%)	674 (52.9%)	10,746 (49.4%)	801 (51.9%)	
Other	4,539 (18.5%)	221 (17.3%)	4,040 (18.6%)	278 (18.0%)	
Unknown	1,203 (4.90%)	76 (5.97%)	1,053 (4.84%)	74 (4.80%)	
Beneficiary Type, n (%)					< 0.01
Active Duty Service Member	6,541 (26.6%)	346 (27.2%)	5,855 (26.9%)	340 (22.0%)	
Family Member	18,016 (73.4%)	928 (72.8%)	15,885 (73.1%)	1,203 (78.0%)	
Substance Use Disorder, n (%)	77 (0.3%)	Low sample size	63 (0.3%)	Low sample size	0.16
Alcohol Use Disorder, n (%)	118 (0.5%)	Low sample size	108 (0.5%)	Low sample size	0.76
Mental Health Diagnosis Pre, n (%)	4,503 (18.3%)	218 (17.1%)	3,880 (17.8%)	405 (26.2%)	< 0.01
Pain Condition Pre, n (%)	8,123 (33.1%)	386 (30.3%)	7118 (32.7%)	619 (40.1%)	< .001
Sexually Transmitted Infection, n (%)	664 (2.7%)	29 (2.3%)	584 (2.7%)	51 (3.3%)	0.26
Preeclampsia, n (%)	1,028 (4.2%)	59 (4.6%)	895 (4.1%)	74 (4.8%)	
Premature Labor, n (%)	52 (0.21%)	Low sample size	40 (0.2%)	Low sample size	0.04
Gestational Diabetes, n (%)	2,826 (11.5%)	124 (9.7%)	2,492 (11.5%)	210 (13.6%)	0.02
Hypertension, n (%)	1,656 (6.7%)	97 (7.61%)	1,439 (6.6%)	120 (7.8%)	0.26
Chorioamnionitis, n (%)	170 (0.69%)	Low sample size	151 (0.7%)	Low sample size	1.00
Single Birth, n (%)	22,928 (93.4%)	1,214 (95.3%)	20,309 (93.4%)	1,405 (91.1%)	< 0.01
Concurrent Tubal Ligation, n (%)	2,668 (10.9%)	145 (11.4%)	2,313 (10.6%)	210 (13.6%)	< 0.01
Non-Opioid Prescription, n (%)	5,818 (23.7%)	285 (22.4%)	5,069 (23.3%)	464 (30.1%)	< 0.01
**Discharge Prescription Information**					
Non-Opioid Prescription	23,467 (95.6%)	729 (57.2%)	21,257 (97.8%)	1,481 (96.0%)	< 0.01
MED, median [IQR]	225 [150;270]		225 [150;300]	225 [150;300]	0.59
Opioid Days Supply, median [IQR]	5.0 [3.0;6.0]		5.0 [3.0;6.0]	5.0 [3.0;6.0]	0.55
Opioid-Only (Non-Combination) Medication, n (%)	4,913 (20.0%)		4,671 (21.5%)	242 (15.7%)	< 0.01
**Post-Cesarean Section Outcomes**					
Post-Partum Depression, n (%)	489 (2.0%)	17 (1.3%)	434 (2.0%)	38 (2.5%)	0.24
Opioid Prescription 30 Days Post-Discharge, n (%)	1,728 (7.0%)	185 (14.5%)	0 (0.00%)	1,543 (100%)	
Opioid Prescription 90 Days Post-Discharge, n (%)	2,334 (9.50%)	199 (15.6%)	592 (2.72%)	1,543 (100%)	

“Low sample size” indicates a cell size was < 10 patients or was in the same row as a cell with low sample size and was removed to obsfucate the distribution

^*^The *p*-value corresponds to bivariate analyses examining the differences between those who did not versus did receive a refill. Continuous variable comparisons analyzed with Kruskall-Wallis tests and are displayed as medians [interquartile ranges]. Categorical variable comparisons analyzed with Chi-square tests and are displayed as frequency (%). *MED* Morphine equivalent dose

In review of the discharge opioid prescription MED, 73% of opioid prescriptions had one of three MEDs: 225 mg (33%), 150 mg (24%), or 300 mg (16%). Approximately 72% of discharge opioid prescriptions had a 5-day supply or less; 94% had a 14-day supply or less. Overall, 15% of the sample received a discharge medication set consistent with CPG and clinical research (no opioid prescription or an opioid-only prescription with a MED < 150 mg; and a separate non-opioid pain medication prescription).

### Contextual-level factors and between-facility variation

**Fig. 2:**
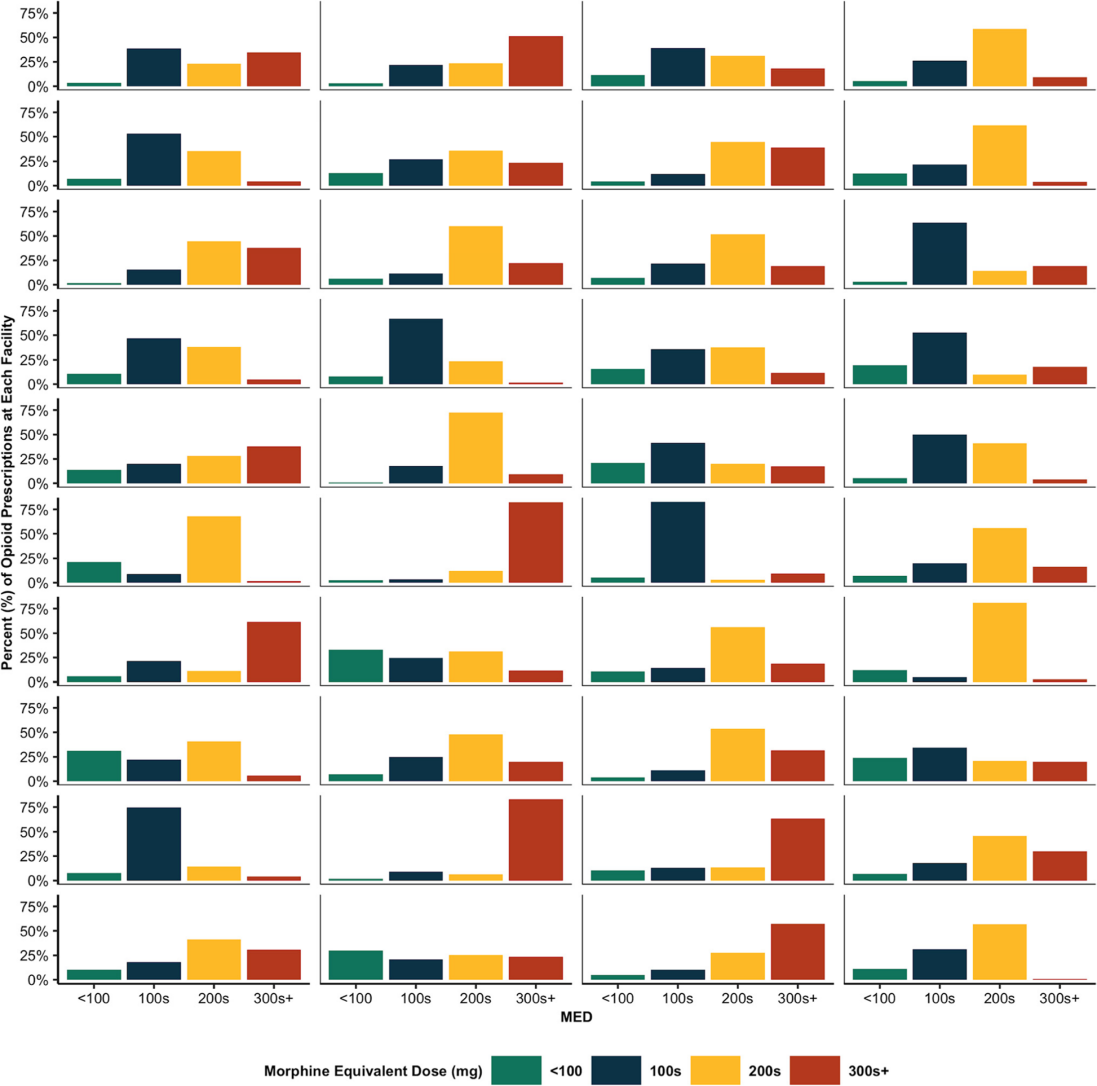
Proportion of Cesarean section discharge opioid prescription MED < 100, 100–199, 200–299, 300 + mg. Note: only facilities with > 124 Cesarean sections during the study period are displayed. Each tile represents one facility

There were 57 facilities represented in the data, 53 of which had at least 10 discharge opioid prescriptions in the study period. In review of facility-level trends, the MED, pill count, and days supply of each discharge prescription was tallied and consolidated. The top three most common discharge MED (150 mg, 225 mg, 300 mg), accounted for 13%—92% of discharge opioid prescriptions across these 53 facilities. Median discharge MED varied across sites; the majority of prescriptions at 28 facilities had a median discharge MED of 225 mg. Whereas, 19 of the 53 facilities had a median discharge MED < 200 mg and 6 facilities had a median discharge MED ≥ 300 mg. Figure 2 provides an overview of MED variation in the 40 highest CS-volume facilities for descriptive and illustrative purposes.

### Bivariate relationships between potential covariates with discharge MED and probability of refill

Non-parametric correlations indicated that discharge MED was not significantly associated with hospitalization duration, but was significantly associated with age (rho = -0.01, *p* < 0.05). Several rank sum tests indicated significant median differences in discharge MED across different categorical factors. Discharge MED significantly varied by documented alcohol use disorder, substance use disorder, mental health condition, pain condition, race and ethnicity (all p ≤ 0.001), and concurrent tubal ligation procedure (*p* = 0.34). The median (225 mg) and lower quartile (150 mg) MED were the same across all pairwise comparisons, minus race and ethnicity. The upper quartile MED (range 225 mg—300 mg) was higher for patients without documented mental health and pain conditions, but with prior documented substance use disorder. In pairwise comparisons of discharge MED and race and ethnicity using false discovery rate-corrected *p*-values, Asian patients had significantly higher discharge MED relative to Black (*p* = 0.01), Latinx (*p* = 0.04), and white (*p* = 0.01) patients; patients whose race and ethnicity was Other had significantly higher discharge MED relative to Black (*p* = 0.03) and white (*p* = 0.04) patients. As indicated in Table 1, there were several significant differences between those who did not and did receive an opioid refill in the first 30 days after CS discharge. Factors significantly associated with discharge MED and refill groups were included as covariates in the GAMM.

### Multilevel model predicting opioid refill

In the GAMM, covariates included age, race and ethnicity, active duty status, hospitalization duration, mental health diagnosis, pain diagnosis, substance use disorder, alcohol use disorder, gestational diabetes, discharge opioid type, concurrent tubal ligation procedure, presence of a single birth, and discharge MED, as well as a random effect for facility. As shown in Table 2, beneficiary type, mental health condition, pain condition, opioid-only discharge prescription, single birth, and age were associated with the probability of opioid refill; whereas race and ethnicity, hospital duration, substance use disorder, alcohol use disorder, gestational diabetes, concurrent tubal ligation, and discharge MED were not.

**Table 2:** Generalized Additive Mixed Model Results Predicting Probability of Opioid Refill

Variable	**Estimate**	**z**	***p*-value**	**Odds Ratio (95% CI)**
(Intercept)	-2.75 (-3.09, -2.41)	-15.95	< 0.001	
Race and Ethnicity (Ref: white)				
Latinx	-0.08 (-0.4, 0.24)	-0.48	0.63	0.92 (1.08, 0.79)
Asian	-0.11 (-0.34, 0.12)	-0.92	0.36	0.9 (0.98, 0.82)
Black	-0.04 (-0.19, 0.11)	-0.45	0.65	0.97 (0.98, 0.95)
Other	-0.07 (-0.22, 0.08)	-0.94	0.35	0.93 (0.96, 0.9)
Unknown	-0.16 (-0.42, 0.1)	-1.22	0.22	0.85 (0.96, 0.75)
Family Member	0.31 (0.17, 0.45)	4.39	< 0.001	1.36 (1.3, 1.43)
Days Hospital Duration	-0.01 (-0.07, 0.05)	-0.41	0.68	0.97 (0.99, 0.96)
Mental Health Condition	0.48 (0.36, 0.6)	7.74	< 0.001	1.62 (1.66, 1.59)
Pain Condition	0.35 (0.24, 0.46)	6.11	< 0.001	1.41 (1.41, 1.42)
Substance Use Disorder	0.52 (-0.2, 1.24)	1.41	0.16	1.68 (2.92, 0.96)
Alcohol Use Disorder	-0.64 (-1.56, 0.28)	-1.37	0.17	0.53 (1.11, 0.25)
Gestational Diabetes	0.08 (-0.08, 0.24)	1.01	0.31	1.08 (1.13, 1.04)
Opioid-Only Discharge Prescription	-0.48 (-0.64, -0.32)	-5.77	< 0.001	0.62 (0.65, 0.59)
Concurrent Tubal Ligation	0.08 (-0.08, 0.24)	0.97	0.33	1.08 (1.14, 1.03)
Single Birth	-0.2 (-0.39, -0.01)	-2.05	0.04	0.82 (0.77, 0.88)
Smooth Terms	edf	Chi-square	*F*-value	
Age	1.84	56.67	< 0.001	
Discharge MED	1	1.71	0.20	

Model included a random effect for facility. Covariates refer to diagnoses and documented information occurring in the 6-months prior to C-section or at discharge

*SE* Standard error, *MED* Morphine Equivalent Dose, *edf* effective degrees of freedom

**Fig. 3:**
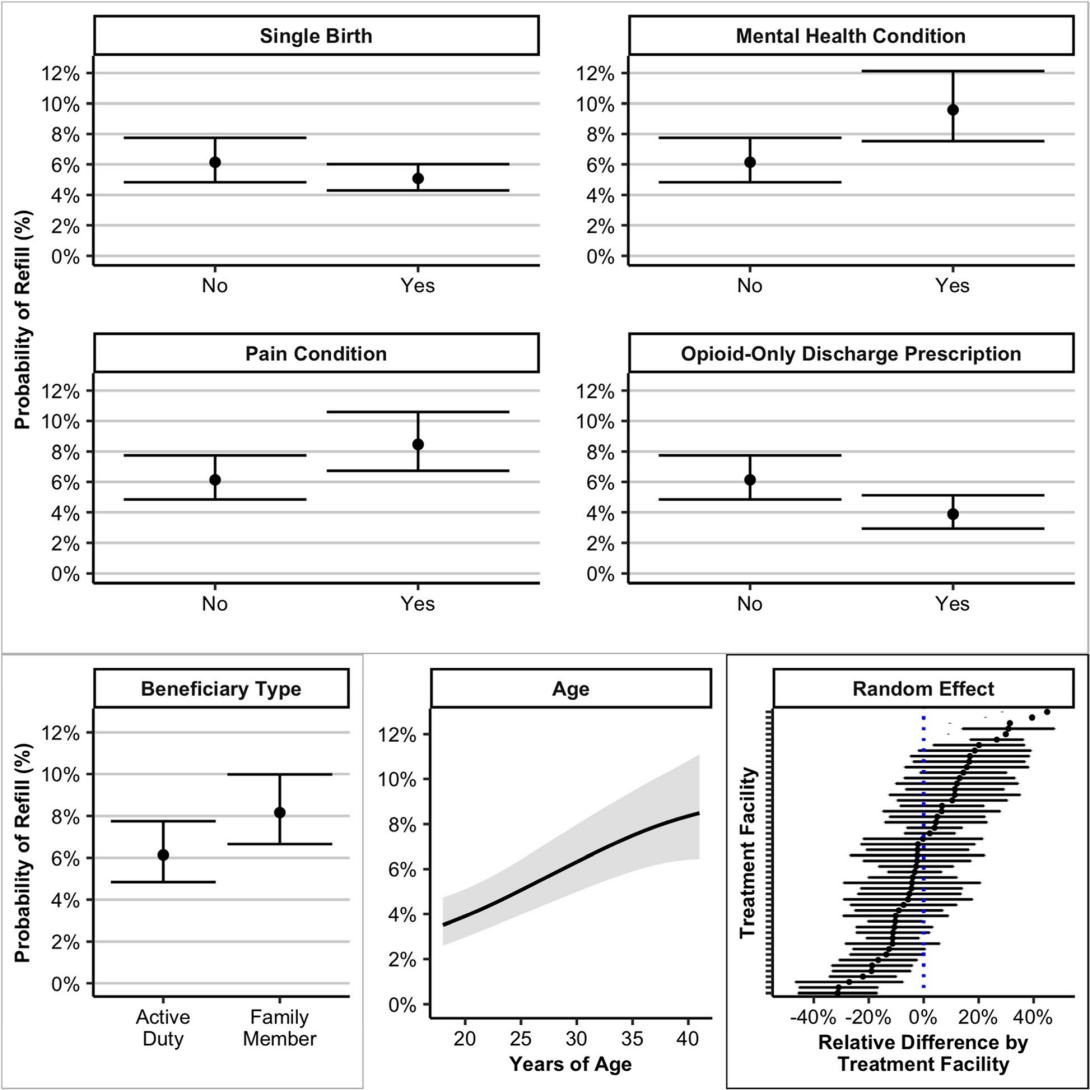
Factors significantly associated with the probability of opioid refill within 30 days after Cesarean section based on results of the generalized additive mixed model

Non-active duty family members, patients with pre-CS mental health and pain conditions, those who received an opioid/non-opioid combination prescription at discharge, and those with multiple births on the day of CS had a higher probability of refill, relative to their counterparts. The smooth term for age indicated that increasing age was associated with increasing probability of opioid refill. Albeit the difference in refill probability for an 18-year-old patient was approximately 4% (95% CI 3%, 5%) and for a 29-year-old patient, 6% (95% CI 5%, 8%), indicating the clinical meaningfulness of difference may be at the margins of age. The marginal effects of significant factors are depicted in Fig. 3.

In sensitivity analyses, patients with pre-CS mental health and pain conditions, and those who received an opioid/non-opioid combination prescription at discharge remained significant predictors and in the same direction in both active duty service members and family members. There was also a significant positive relationship between age and probability refill across both models, similar to the overall model. However, single birth CS were significantly associated with the probability of refill in Family Members (*p* = 0.02), but not in Active Duty patients (*p* = 0.83). Taken together, sensitivity models indicated congruence with the full model results, minus the exception of single birth deliveries.

## Discussion

### Principal findings

In this analysis of patients undergoing CS procedures in the MHS, we found a significant variation in discharge opioid MED and opioid medication type (e.g., opioid-only versus combined opioid/non-opioid medication). Moreover, variation in prescriptions occurred within facilities, with some facilities showing a range of discharge opioid MEDs, while others showing modal use of a single discharge opioid MED, suggestive of a default prescription order set. These results are consistent with previous literature regarding variation in post-procedural opioid prescribing practices by state, such that variation in opioid dose receipt could be explained, in part, by larger contextual factors (e.g., facility) [17, 18]. The multivariable model indicated that discharge opioid prescription’s MED was not associated with the probability of an opioid refill, but the opioid type was.

## Results

Multivariable model results indicated that, when controlling for other factors, patients who received an opioid/non-opioid combination medication were more likely to obtain an opioid prescription refill than those who received an opioid-only medication. Though both opioid and non-opioid pain medications are part of the ACOG guidelines—to include “opioids that are in combination formulations with either acetaminophen or an NSAID,” [6] the results of the present study suggest prescribing the opioid and non-opioid medications separately may be helpful in providing patients greater flexibility in medication dosing and addressing their pain management needs, thereby reducing the need for more opioids and a subsequent refill. Such a prescribing approach may be most effective when paired with shared decision making and patient education regarding the utility and purpose of opioid versus non-opioid medications. For example, most patients may obtain adequate pain relief when receiving 20 pills or less [8]; and when provided the education and agency to make a decision, most patients estimate that 20 pills would be adequate after CS birth [19].

### Clinical implications

Though the ACOG guidelines indicate that providers need to work with patients to best tailor post-discharge pain management plans, this does not eliminate the need to reconsider and examine default order sets. In the present study, the predominant utilization of three order sets suggest an elevated reliance on default opioid prescription orders. Previous studies have shown that changing the post-surgical discharge default order sets can significantly reduce excessive opioid prescribing practices [20]. Thus, electronic health records and embedded decision support tools may benefit from implementation of a multimodal post-CS discharge order set, to include non-opioid pain medications (e.g., NSAIDs, acetaminophen) and an option to add an opioid-only medication. Future work is needed to ensure any automated order set and clinical decision support tool is evidence-based, efficient, and supportive of but not a replacement to shared decision making.

### Research implications

The present study did not include variables relevant to the inpatient analgesia pathway (e.g., use of general anesthesia, transfusion needs, inpatient opioid use and non-opioid use during hospitalization, etc.) or utilization of Enhanced Recovery After Cesarean (ERAC) [21] protocols. ERAC protocol implementation may reduce the need for opioid-based analgesia both while inpatient and after discharge [22], as well as costs [23]. Variable implementation of ERAC protocols could explain the variation in discharge medication prescriptions, including the finding that 5% of the sample was discharged without an opioid prescription, but this proportion varied across facilities (from 1 to 28% of patients were discharged without an opioid prescription). In our previous analysis, we found that inpatient opioid receipt and additional inpatient analgesia pathway components and patient-reported pain intensity while inpatient were not associated with discharge MED at a large military treatment facility [24]. Given that such factors could be associated with post-discharge opioid utilization, future work is needed to include inpatient analgesia pathway components and patient-reported outcomes (e.g., pain management satisfaction, opioid utilization).

### Strengths and limitations

In the present study, the utilization of MHS data provided the opportunity to examine healthcare variation across dozens of healthcare facilities. Findings illustrated both healthcare variation and factors associated with such variation, including the notion that variation needs to be addressed at the facility-level (as variation persisted despite all facilities being part of the the same healthcare system). Analyses leveraged a GAMM, an analytic approach that allowed for the evaluation of non-normally distributed predictors, outcomes, and relationships, as well as the inclusion of random effects (e.g., treatment facility). In doing so, the present findings are less subject to the pitfalls of parametric and simple linear regressions, while also providing a more robust multivariable analysis of a wide variety of predictors. Lastly, the inclusion of sensitivity analyses indicated that findings are largely consistent across active duty and family member beneficiaries.

In regards to limitations, the generalizability of the present study is limited to patients who received either no opioid prescription or a single opioid prescription at discharge, had a hospital duration < 8 days, and were opioid naive in the 6-months preceding the CS. It is unclear how the present findings would replicate or differ in a sample of patients who underwent vaginal delivery or had pregnancy-related complications that required prolonged hospitalization. Generalizability may also be limited by the underlying patient population of active duty service members and their family members who were continuously insured in the 6-months preceding and following their CS birth. Lastly, it is unclear the degree to which the United States opioid prescribing practices after CS discharge are paralleled to other countries. Previous evidence indicates that patients undergoing common surgeries in the United States are significantly more likely to receive an opioid prescription at discharge (91%) and a refill (5%), relative to patients in other countries (5 and 1%, respectively) [25]. As such, the present findings may not generalize to healthcare systems outside of the United States.

Given that CS outcomes may vary due to systemic inequities in healthcare access and quality [26, 27], greater care is needed to evaluate not only variation and outcomes related to opioid use, but additional outcomes associated with quality of life, chronic pain, and overall functioning. There was also a lack of information regarding other outcomes, such as return to duty or work, that may be meaningful to explore in future studies. As indicated previously, the data did not include information regarding the analgesia pathway, including ERAC utilization, nor were inpatient pain scores available for inclusion in our model, which may be especially important to consider given the variability in pain experiences and their relationships with opioid utilization [28, 29]. Future studies will benefit from implementing and evaluating process metrics related to ERAC implementation. The flags for mental health and pain conditions are not all-encompassing, and instead, represent common Axis I mental health conditions, per the Diagnostic and Statistical Manual V, and musculoskeletal conditions that were largely acute in nature (e.g., lumbago and other spine-related conditions). Given the degree to which diagnostic conclusions and medical coding can vary, the selected broad categories were meant to provide some indication that either types of conditions were relevant to further analysis.

## Conclusions

Significant variation in discharge pain medication prescriptions, as well as the lack of association between discharge opioid MED and probability of refill, indicates that efforts are needed to optimize opioid prescribing and reduce unnecessary healthcare variation. ERAC protocols, decision support tools, interventions for shared decision making, and default order sets may benefit from an all-encompassing integrative evaluation and restructure to not only reduce variation, but to ensure patient-centered pain management after CS.

## Supplementary Information

**Additional file 1:** **Supplemental Figure 1.** Variation in the proportion of patients who did not receive an opioid prescription at discharge. Each bar represents a different Military Health System facility. The figure depicts 53 of the 57 facilities with at least 10 discharge opioid prescriptions.

## Data Availability

All data generated or analyzed during this study are not included in this published article. The data is sourced from the Department of Defense and cannot be made available directly to the public or individual researchers by the authors. Any request for data would need to go through the appropriate procedures (e.g., data sharing agreements) and authorities (Defense Health Agency).

## Abbreviations

MED: Morphine equivalent dose
GAMM: Generalized additive mixed model
CS: Cesarean section
ACOG: American College of Obstetricians and Gynecologists
NSAIDS: Non-steroidal anti-inflammatory drugs
MHS: Military health system
MEQ: Morphine equivalence
ERAC: Enhanced Recovery After Cesarean
