# Supplementary material for: Variation by default: cesarean section discharge opioid prescription patterns and outcomes in Military Health System hospitals: a retrospective longitudinal cohort study
Source: BMC Anesthesiol. 2022 Jul 12;22:218. doi: 10.1186/s12871-022-01765-8 (PMC9277874; doi:10.1186/s12871-022-01765-8)
Supplement: Supplementary file 1 — Additional file 1: Supplemental Figure 1. Variation in the proportion of patients who did not receive an opioid prescription at discharge. Each bar represents a different Military Health System facility. The figure depicts 53 of the 57 facilities with at least 10 discharge opioid prescriptions. [file 12871_2022_1765_MOESM1_ESM.pdf]

Table 1. Descriptive statistics of the full sample, those who did not receive an opioid at discharge, and those who did not and did refill an opioid prescription within 30 days after discharge.

	Full Sample (N = 24,557)	No Discharge Opioid n = 1,274 (5.2%)	No Refill* n = 21,740 (88.5%)	Refill* n = 1,543 (6.3%)	p-value*
**Demographic and Pre-Cesarean-Section Factors**					
Age, median years [IQR]	28.0 [24.0;32.0]	29.0 [24.0;33.0]	28.0 [24.0;32.0]	29.0 [25.0;33.0]	<0.01
Race and Ethnicity, n (%)					0.62
Latinx white	851 (3.5%)	31 (2.4%)	774 (3.6%)	46 (3.0%)	
Non-Latinx Asian	1,437 (5.9%)	66 (5.2%)	1,280 (5.9%)	91 (5.9%)	
Non-Latinx Black	4,306 (17.5%)	206 (16.2%)	3,847 (17.7%)	253 (16.4%)	
Non-Latinx white	12,221 (49.8%)	674 (52.9%)	10,746 (49.4%)	801 (51.9%)	
Other	4,539 (18.5%)	221 (17.3%)	4,040 (18.6%)	278 (18.0%)	
Unknown	1,203 (4.90%)	76 (5.97%)	1,053 (4.84%)	74 (4.80%)	
Beneficiary Type, n (%)					<0.01
Active Duty Service Member	6,541 (26.6%)	346 (27.2%)	5,855 (26.9%)	340 (22.0%)	
Family Member	18,016 (73.4%)	928 (72.8%)	15,885 (73.1%)	1,203 (78.0%)	
Substance Use Disorder, n (%)	77 (0.3%)	Low sample size	63 (0.3%)	Low sample size	0.16
Alcohol Use Disorder, n (%)	118 (0.5%)	Low sample size	108 (0.5%)	Low sample size	0.76
Mental Health Diagnosis Pre, n (%)	4,503 (18.3%)	218 (17.1%)	3,880 (17.8%)	405 (26.2%)	<0.01
Pain Condition Pre, n (%)	8,123 (33.1%)	386 (30.3%)	7118 (32.7%)	619 (40.1%)	<.001
Sexually Transmitted Infection, n (%)	664 (2.7%)	29 (2.3%)	584 (2.7%)	51 (3.3%)	0.26
Preeclampsia, n (%)	1,028 (4.2%)	59 (4.6%)	895 (4.1%)	74 (4.8%)	
Premature Labor, n (%)	52 (0.21%)	Low sample size	40 (0.2%)	Low sample size	0.04
Gestational Diabetes, n (%)	2,826 (11.5%)	124 (9.7%)	2,492 (11.5%)	210 (13.6%)	0.02
Hypertension, n (%)	1,656 (6.7%)	97 (7.61%)	1,439 (6.6%)	120 (7.8%)	0.26
Chorioamnionitis, n (%)	170 (0.69%)	Low sample size	151 (0.7%)	Low sample size	1.00
Single Birth, n (%)	22,928 (93.4%)	1,214 (95.3%)	20,309 (93.4%)	1,405 (91.1%)	<0.01
Concurrent Tubal Ligation, n (%)	2,668 (10.9%)	145 (11.4%)	2,313 (10.6%)	210 (13.6%)	<0.01
Non-Opioid Prescription, n (%)	5,818 (23.7%)	285 (22.4%)	5,069 (23.3%)	464 (30.1%)	<0.01
**Discharge Prescription Information**					
Non-Opioid Prescription	23,467 (95.6%)	729 (57.2%)	21,257 (97.8%)	1,481 (96.0%)	<0.01
MED, median [IQR]	225 [150;270]		225 [150;300]	225 [150;300]	0.59
Opioid Days Supply, median [IQR]	5.0 [3.0;6.0]		5.0 [3.0;6.0]	5.0 [3.0;6.0]	0.55
Opioid-Only (Non-Combination) Medication, n (%)	4,913 (20.0%)		4,671 (21.5%)	242 (15.7%)	<0.01
**Post-Cesarean Section Outcomes**					
Post-Partum Depression, n (%)	489 (2.0%)	17 (1.3%)	434 (2.0%)	38 (2.5%)	0.24
Opioid Prescription 30 Days Post-Discharge, n (%)	1,728 (7.0%)	185 (14.5%)	0 (0.00%)	1,543 (100%)	
Opioid Prescription 90 Days Post-Discharge, n (%)	2,334 (9.50%)	199 (15.6%)	592 (2.72%)	1,543 (100%)	

Note: “Low sample size” indicates a cell size was <10 patients or was in the same row as a cell with low sample size and was removed to obsfucate the distribution. *The p-value corresponds to bivariate analyses examining the differences between those who did not versus did receive a refill. Continuous variable comparisons analyzed with Kruskall-Wallis tests and are displayed as medians [interquartile ranges]. Categorical variable comparisons analyzed with Chi-square tests and are displayed as frequency (%). MED = morphine equivalent dose
